# Discharge Practices After Hospitalization for COPD Exacerbations: A Physician Survey and SWOT Analysis

**DOI:** 10.3390/healthcare14121786

**Published:** 2026-06-20

**Authors:** Sanja Dimic-Janjic, Mihailo Stjepanovic, Ivan Cekerevac, Sanja Hromis, Ivana Buha, Vojislav Cupurdija, Ivan Kopitovic, Rade Milic, Biljana Zvezdin, Ivana Stankovic, Jelena Jankovic, Nikola Trboljevac, Maja Omcikus, Lidija Isovic, Nikola Kostadinovic, Nikola Subotic, Marija Vukoja

**Affiliations:** 1 Faculty of Medicine, University of Belgrade,11000 Belgrade, Serbia; 2 Clinic for Pulmonology, University Clinical Center of Serbia, 11000 Belgrade, Serbia; 3 Faculty of Medicine, University of Kragujevac, 34000 Kragujevac, Serbia; 4 Clinic for Pulmonology, University Clinical Center of Kragujevac, 34000 Kragujevac, Serbia; 5 Faculty of Medicine, University of Novi Sad, 21000 Novi Sad, Serbiamarija.vukoja@mf.uns.ac.rs (M.V.); 6 Institute for Pulmonary Diseases of Vojvodina, 21000 Novi Sad, Serbia; 7 Medical Faculty of the Military Medical Academy, University of Defense in Belgrade, 11000 Belgrade, Serbia; 8 Clinic for Pulmonology, Military Medical Academy, 11000 Belgrade, Serbia; 9 Faculty of Medicine, University of Nis, 18000 Nis, Serbia; 10 Clinic for Pulmonology, University Clinical Center of Nis, 18000 Nis, Serbia

**Keywords:** chronic obstructive pulmonary disease, COPD, hospital discharge, transitional care, discharge planning, implementation gap

## Abstract

**Highlights:**

**What are the main findings?**

- COPD discharge practices in Serbia show substantial variability and limited standardization despite broad awareness of recommended care principles.
- Physicians reported high willingness to adopt structured discharge tools despite their limited routine use in clinical practice.

**What are the implications of the main findings?**

- The findings highlight discharge standardization, care coordination, and patient education as potential targets for future quality-improvement initiatives.
- The high level of physician support for structured discharge tools suggests that organizational factors may represent important barriers to implementation.

**Abstract:**

Background/Objectives: Discharging patients after hospitalization for an acute exacerbation of chronic obstructive pulmonary disease (COPD) is a critical transition in care associated with a high risk of early readmission. This survey aimed to describe physician-reported discharge practices following COPD exacerbations, identify perceived gaps and organizational barriers, explore attitudes toward structured COPD discharge summaries, and use a SWOT analysis as an interpretative framework. Methods: In this cross-sectional observational survey, 100 physicians involved in COPD care were recruited from the official mailing list of the Respiratory Society of Serbia, which represents approximately 71% of the Society’s members. The survey assessed discharge procedures, multidisciplinary practices, patient education, comorbidity management, perceived causes of readmission, and barriers to structured discharge summaries. Data were analyzed descriptively and complemented with a structured SWOT (Strengths, Weaknesses, Opportunities, Threats) analysis. Results: Most respondents worked in tertiary care settings and were involved in managing patients hospitalized for COPD exacerbations. Although 24% of physicians routinely used structured discharge summaries, 45% reported never using them. The most frequently perceived contributors to 30-day readmissions were active smoking (90%), poor treatment adherence (81%), comorbidities (77%), and incorrect inhaler technique (72%). Major barriers to implementing structured discharge summaries included the lack of standardized templates, time constraints, poor coordination across healthcare levels, and technical limitations. Willingness to implement structured discharge tools was high (mean score 8.86/10). SWOT analysis identified strong professional support for discharge standardization alongside organizational and system-level barriers to implementation. Conclusions: This exploratory survey identified important gaps between recommended and routine COPD discharge practices and highlighted organizational barriers to implementation. The findings may inform future evaluation and development of structured discharge tools in this healthcare setting.

## 1. Introduction

Acute exacerbations of chronic obstructive pulmonary disease (COPD) are a significant cause of hospitalization, morbidity, and healthcare utilization [1,2,3,4]. The transition from hospital to outpatient care represents a critical phase in COPD management [5,6], during which inadequate communication, incomplete discharge documentation, and insufficient patient education may contribute to poor outcomes and early readmissions [7]. Up to a quarter of patients hospitalized for an exacerbation are readmitted within 30 days of discharge [8], and 40% of patients are readmitted or die within 90 days of discharge [9]. Despite existing recommendations for comprehensive COPD discharge planning, discharge practices in real-world settings remain inconsistent and lack standardization. International guidelines, including the GOLD report, recommend structured discharge planning that incorporates treatment optimization, patient education, comorbidity assessment, and follow-up care [10]. More recently, COPD management has increasingly emphasized organized discharge bundles and post-exacerbation care pathways rather than discharge documentation alone. These multidimensional interventions typically include treatment optimization, inhaler technique assessment, smoking cessation support, pulmonary rehabilitation referral, comorbidity management, patient education, and structured follow-up arrangements. Evidence suggests that such coordinated approaches may improve continuity of care and reduce the risk of readmission following hospitalization for COPD exacerbation. Within this framework, structured discharge summaries may serve as an important tool to facilitate communication and ensure consistent implementation of recommended post-discharge interventions [10,11].

However, the extent to which these recommendations are implemented in routine clinical practice remains unclear. Structured COPD discharge bundles and discharge documentation tools have been developed and evaluated in several healthcare systems, including national audit programs, consensus initiatives, and quality-improvement projects. For example, national COPD audit programs in the United Kingdom have demonstrated substantial variability in the implementation of discharge bundle elements [12], while structured discharge recommendations have recently been formalized through national consensus initiatives in Poland [13]. Furthermore, a recent study from neighboring Croatia identified major deficiencies in guideline-concordant COPD discharge documentation, highlighting persistent gaps between recommendations and routine clinical practice [14]. Also, national data describing COPD discharge practices, implementation barriers, and readiness to adopt structured discharge tools in Serbia are lacking, and evidence from middle-income healthcare systems in Eastern Europe remains limited.

To our knowledge, this is the first study to systematically evaluate physician-reported COPD discharge practices, implementation barriers, and readiness to adopt structured discharge summaries within a respiratory professional network. By combining a physician survey with SWOT analysis, the study provides both a descriptive overview of current practice and an interpretative framework for understanding organizational barriers and implementation challenges. The study was designed to explore physician-reported discharge practices, perceived gaps, organizational barriers, and readiness to adopt structured discharge summaries. By identifying areas of variability and implementation challenges, the findings may help inform future quality-improvement efforts and the evaluation of structured discharge tools within this healthcare setting.

## 2. Materials and Methods

### 2.1. Study Design, Participants, and Recruitment

We conducted a cross-sectional observational survey to describe physician-reported discharge practices following COPD exacerbations, identify perceived gaps and organizational barriers, explore attitudes toward structured COPD discharge summaries, and use a SWOT analysis as an interpretative framework to understand factors influencing discharge practices.

The target group included pulmonologists, internal medicine specialists, and internal medicine residents working in pulmonary departments. In the Serbian healthcare system, the targeted physician population is primarily responsible for managing hospitalized patients with COPD exacerbations and routinely provides care for patients across all stages of COPD in outpatient clinics, emergency departments, and respiratory intensive care units. Participants were invited via email through the official mailing list of the Respiratory Society of Serbia. Although the survey invitation was distributed through the Society mailing list, responses were collected anonymously using Microsoft Forms (Microsoft Corporation, Redmond, WA, USA). Consequently, individual participant identities could not be linked to survey responses. At the time of the survey, the Society included approximately 140 members, predominantly pulmonologists, internal medicine specialists working in pulmonary departments, and residents working in pulmonary departments. The survey remained open for 20 days (26 December 2025–15 January 2026). A reminder email was distributed 10 days after the initial invitation to improve participation.

At the end of the survey period, 100 complete questionnaires were available for analysis, corresponding to approximately 71% of the Society membership. The survey was conducted electronically using Microsoft Forms. Participation was entirely voluntary, and no incentives were provided.

Given the relatively high participation rate, the survey provides a useful overview of current clinical practice patterns among physicians involved in COPD care.

### 2.2. Questionnaire Development and Validity

The survey questionnaire was developed explicitly for this study using a structured, multistep process. Initial items were generated by a core group of pulmonologists based on current clinical guidelines, published literature on COPD discharge and transitional care, and routine clinical practice [15,16]. Content validity was established during a dedicated expert meeting that included 13 pulmonologists and clinicians experienced in the inpatient management of COPD and discharge planning. During this meeting, questionnaire items were systematically reviewed and discussed to ensure comprehensive coverage of all clinically relevant domains of the COPD discharge process, including clinical stability, pharmacological management, comorbidity assessment, patient education, follow-up planning, and care coordination. Items were evaluated for relevance, completeness, and appropriateness for the target clinical setting.

Following the expert meeting, the questionnaire underwent iterative refinement via email-based review. This process enabled additional expert feedback on item wording, structure, and clarity, resulting in minor modifications to improve consistency and usability while preserving the original content domains.

Face validity was supported through expert assessment of the clarity, interpretability, and clinical relevance of individual items. Formal psychometric validation was not conducted because the study’s primary aim was to provide a descriptive assessment of current practice and perceived needs, rather than to develop a quantitative measurement scale.

The complete questionnaire, including all items and response options, is provided as online data in Supplementary File S1. The questionnaire was initially developed and administered in Serbian and subsequently translated into English for publication. The questionnaire consisted of 20 items organized into five thematic domains: respondent characteristics and practice setting (2 items), current discharge practices and organizational aspects (8 items), patient education and self-management (3 items), readmissions and perceived outcomes (3 items), and structured discharge summaries, implementation barriers, and future adoption (4 items). The questionnaire included 12 Likert-scale items, 7 multiple-choice questions, and one 0–10 numerical rating-scale item assessing the likelihood of future use of structured discharge summaries. No formal pilot testing was performed prior to questionnaire dissemination.

### 2.3. Survey Instrument

A discharge summary is defined as an important clinical document that accurately and succinctly describes the patient’s medical history, diagnoses, treatment, and follow-up plans during hospital admission [17].

The final structured questionnaire consisted of five thematic domains:

1. Current discharge practice, including level of healthcare institution, professional background of respondents, multidisciplinary team involvement, use and frequency of structured discharge summaries, standardization of discharge procedures, and the presence of a designated discharge coordinator.
2. Perceived gaps and challenges, focusing on patient stability assessment before discharge, clarity of discharge therapy, follow-up planning, communication between levels of care, and consideration of comorbidities.
3. Patient education and self-management, including inhaler technique training, patient understanding of discharge instructions, and provision of written action plans for future exacerbations.
4. Outcomes and readmissions, addressing perceived rates of 30-day readmissions and factors contributing to rehospitalization.
5. Interest in and justification for a structured discharge protocol, including perceived benefits, essential components of a standardized discharge summary, likelihood of future use, and barriers to implementation.

Responses were collected using five-point Likert scales (ranging from strong disagreement to strong agreement), frequency scales (ranging from never to always), and a numerical probability scale (0–10). Several items allowed multiple-choice responses and optional open-ended responses. The average completion time for the questionnaire was 8 min and 42 s. Microsoft Forms settings allowed only one response per participant, preventing duplicate submissions.

The study was reported in accordance with recommended practices for survey-based research.

### 2.4. Ethical Considerations

The survey did not collect any personal identifiers or patient-level data. Anonymity and confidentiality of responses were fully ensured.

At the beginning of the questionnaire, participants were provided with detailed information regarding the study objectives, the voluntary nature of participation, and the right to withdraw at any time without consequences. The continuation and completion of the survey implied informed consent. The study was approved by the Ethics Committee of the University Clinical Center of Serbia (approval number: 1880/36; date: 25 December 2025).

### 2.5. Statistical Analysis

Data were analyzed using descriptive statistical methods. Categorical variables were summarized as absolute and relative frequencies (counts and percentages), while ordinal variables were presented as distributions across response categories. For questions with multiple response options, percentages were calculated based on the total number of respondents and may exceed 100%. The likelihood of using a structured COPD discharge summary was assessed using a numerical rating scale from 0 (not likely at all) to 10 (extremely likely). Only complete questionnaires were included in the final analysis. Because all analyzed questionnaires were complete, no imputation procedures for missing data were required. Responses on the 0–10 likelihood scale were treated as continuous variables and summarized using the arithmetic mean, standard deviation (SD), median, and interquartile range (IQR). No weighting or transformation was applied. Any subgroup analyses by institution type or physician profile were considered exploratory and performed to identify potential patterns in responses rather than to test prespecified hypotheses.

A SWOT (Strengths, Weaknesses, Opportunities, and Threats) analysis was performed to complement the quantitative findings and provide a structured, semi-qualitative evaluation of factors influencing COPD discharge practices. Internal factors (strengths and weaknesses) were defined as characteristics of current clinical practice identified through survey responses. In contrast, external factors (opportunities and threats) reflected system-level, organizational, and healthcare structure-related influences. The analysis was based on aggregated survey results across key domains, including multidisciplinary team involvement, discharge standardization, patient education, comorbidity assessment, and post-discharge follow-up planning. Survey findings were synthesized and mapped into SWOT categories through iterative consensus among the authors. Discrepancies were resolved through discussion until agreement was reached. The incorporation of SWOT analysis enabled a comprehensive interpretation of the results beyond descriptive statistics, facilitating the identification of implementation gaps and informing potential strategies for improving the quality, consistency, and safety of COPD discharge processes. Given the descriptive nature of the study, no inferential statistical testing was performed. All analyses were performed on aggregated data and used exclusively for research and publication purposes. The statistical analysis was performed using IBM SPSS version 29.

## 3. Results

A total of 100 physicians completed the 20-question survey, with an average completion time of 8 min and 42 s.

### 3.1. Respondent Characteristics

The participants included a diverse group of respiratory professionals, representing a wide range of expertise and experience. The largest groups were subspecialist pulmonologists and phthisiologists (43%), internal medicine specialists (22%), internal medicine residents (26%), and clinical junior GPs working in the pulmonology department (9%). Most participants worked in tertiary healthcare institutions (75%), while smaller proportions worked in secondary care (20%) and private practice (5%).

### 3.2. Multidisciplinary Team Involvement

Regarding who is usually involved in preparing a COPD patient for discharge from the hospital, responses indicate that care for hospitalized patients is primarily provided by physicians, with limited routine participation from a multidisciplinary team (98 specialists, 72 residents, 45 junior clinical physicians, 38 nurses, 30 respiratory therapists, 6 social workers, and 3 pharmacists).

### 3.3. Use of Structured Discharge Summaries, Standardization, and Coordination of Discharge Planning

Implementation of a structured discharge summary after hospitalization for COPD exacerbation is inconsistent, with wide variation in discharge practices (45% reported never using a structured discharge summary, 24% always using one, 15% “rarely,” 5% “sometimes,” and 11% “often”). Regarding uniformity in discharge procedures and summaries across respondents’ institutions or teams, 21% said these are always standardized, 32% often, 22% occasionally, 15% rarely, and 10% reported that discharge processes are never standardized. Variability in discharge standardization across institutions was observed. A designated discharge coordinator was reported as always present by 19% of respondents and often present by 12%, whereas 34%, 25%, and 10% reported no, rare, or occasional presence, respectively.

### 3.4. Challenges Affecting Safe Discharge

The most frequently reported challenges affecting safe discharge are summarized in Table 1. Preventive measures and smoking cessation counseling, communication between healthcare levels, and comorbidities/polypharmacy were among the most reported challenges.

### 3.5. Consideration of Comorbidities

Cardiovascular comorbidities (arterial hypertension, coronary artery disease, heart failure, arrhythmias, pulmonary hypertension, peripheral arterial disease) are generally considered at the discharge of COPD patients, reported as always by 51%, often by 31%, occasionally by 15%, rarely by 2%, and never by 1%. Other common comorbidities in COPD patients (lung cancer, osteoporosis, anxiety/depression, sarcopenia, GERD, metabolic diseases) are consistently considered by 37% of respondents, 34% as often, 20% occasionally, 8% rarely, and 1% never.

### 3.6. Follow-Up Plans, Patient Education, and Self-Management Support

Follow-up, patient education, and self-management practices are summarized in Table 2. Follow-up arrangements and inhaler education were reported more frequently than the provision of written action plans.

### 3.7. Readmissions, Perceived Impact of Structured Discharge, and Factors Associated with Readmission

Responses regarding early readmissions varied. Only 23% of respondents agreed, and 3% strongly agreed, that 30-day rehospitalizations are common. Meanwhile, 36% of respondents were neutral on the issue. In contrast, 37% disagreed, and 1% strongly disagreed. More than half (52%) of respondents agreed that a structured discharge summary can help reduce rehospitalizations after a COPD exacerbation. Additionally, 20% strongly agreed, 20% were neutral, 7% disagreed, and 1% strongly disagreed. Factors perceived to contribute to COPD readmission following hospitalization for exacerbation are summarized in Table 3. Active smoking, poor treatment adherence, comorbidities, and incorrect inhaler technique were the most frequently reported contributors.

### 3.8. Perceived Impact of Structured Discharge on Quality of Care and Essential Components

Most respondents expressed positive views on the idea that a structured discharge summary after a COPD exacerbation would enhance the quality of care. Specifically, 47% agreed, 28% strongly agreed, 23% were neutral, and only 2% disagreed. Respondents outlined components that should be included in a structured discharge summary in Table 4.

### 3.9. Likelihood of Implementing a Structured Discharge Summary and Barriers to Implementation

**Figure 1:**
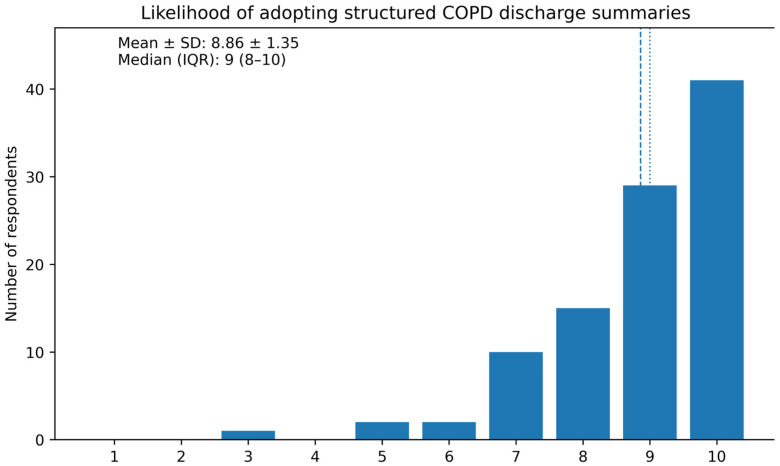
Distribution of physicians’ responses regarding the likelihood of adopting structured COPD discharge summaries in routine clinical practice using a 10-point numerical rating scale (1 = not likely at all; 10 = extremely likely). The mean score was 8.86 ± 1.35, and the median score was 9 (IQR 8–10). The dashed vertical lines indicate the mean (8.86) and median (9) likelihood scores.

Exploratory subgroup analyses by institution type and physician profile showed a consistently high willingness to implement structured discharge summaries across all respondent groups, with mean scores ranging from 8.8 to 9.2. The mean likelihood score for using a structured COPD discharge summary in clinical practice was 8.86 ± 1.35. The median score was 9 (IQR 8–10), indicating a high overall willingness to adopt structured discharge summaries in routine care, as shown in Figure 1.

Respondents’ impressions of the main barriers to implementing a structured discharge summary are shown in Table 5.

**Figure 2:**
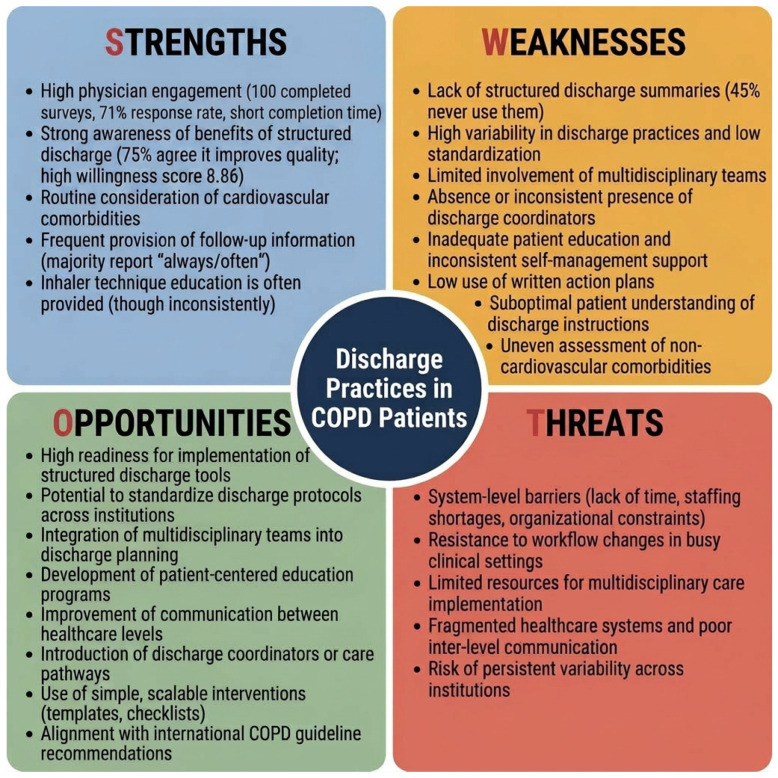
A SWOT (Strengths, Weaknesses, Opportunities, and Threats) analysis. Strengths included high physician awareness of the importance of structured discharge and frequent follow-up planning. Weaknesses included low use of structured discharge summaries, variability in practice, limited multidisciplinary involvement, and inconsistent patient education. Opportunities were identified in the high willingness to adopt structured discharge tools and in the potential for standardization through templates and improved care coordination. Threats were mainly related to system-level barriers, including time constraints, organizational challenges, and poor inter-level communication.

A SWOT (Strengths, Weaknesses, Opportunities, and Threats) analysis was conducted to summarize key findings on COPD discharge practices (Figure 2).

## 4. Discussion

This study provides a real-world overview of COPD discharge practices and reveals substantial variability, limited standardization, and a clear gap between recommended and implemented care. The novelty of this study lies not only in describing current discharge practices but also in identifying implementation gaps and translating these findings into a practical implementation framework through SWOT analysis.

A key finding is a significant implementation gap: although physicians recognize the importance of structured discharge processes and are highly willing to adopt them, these practices are not consistently applied in routine clinical settings. This discrepancy suggests that the main barrier is not a lack of awareness, but rather system-level and organizational constraints.

The SWOT analysis supports this interpretation. While strengths such as high physician engagement and awareness of the importance of discharge are evident, critical weaknesses persist, including low use of structured discharge summaries, inconsistent patient education, and limited multidisciplinary involvement. Similar findings have been reported previously, with studies showing that many hospitals lack formalized discharge processes and standardized discharge tools (Scullion et al., 2010) [18].

System-level barriers appear to play a dominant role in limiting implementation. Time constraints, fragmented communication between healthcare levels, and insufficient organizational support were frequently identified in our survey and are consistent with prior observations (Yeh et al., 2024) [19]. In addition, the importance of patient education and comprehensive care planning has been emphasized in previous studies, highlighting the need for individualized and multidisciplinary approaches (Bourbeau et al., 2020) [20].

Importantly, evidence suggests that structured discharge interventions can significantly improve outcomes. Implementation of standardized discharge protocols and care bundles has been associated with reductions in readmission rates and improvements in care quality (Gupta et al., 2023) [21]. These findings align with international guideline recommendations, including the GOLD report and structured discharge frameworks (Miravitlles et al., 2023), which emphasize treatment optimization, patient education, and continuity of care [11]. Our findings are consistent with observations from other healthcare systems, where substantial gaps between guideline recommendations and routine clinical practice have also been reported. The European COPD Audit (Hartl et al., 2014) demonstrated considerable variability across participating countries in adherence to recommended COPD care processes and in the availability of post-discharge support services [22]. Similarly, recent UK National Respiratory Audit Program (Rashid et al., 2024) data showed that fewer than one-third of hospitalized COPD patients received all recommended discharge bundle elements despite the existence of a formal national quality improvement program [23]. Studies from Croatia (Vukić Dugac et al., 2023) [14] and the United States have also reported low rates of documented inhaler education and structured discharge communication. Together, these findings suggest that challenges related to discharge standardization and implementation are not unique to a single healthcare system but represent a broader international issue. The identified barriers, particularly the lack of standardized discharge tools, limited time, and challenges in coordination between healthcare levels, may be especially relevant in middle-income healthcare systems, where resource constraints often complicate the implementation of comprehensive transitional care programs.

Current GOLD recommendations emphasize that discharge after COPD exacerbation should not be limited to clinical stabilization alone. Still, they should also include optimization of maintenance therapy, confirmation of proper inhaler technique, smoking cessation counseling, provision of a written self-management/action plan, and early post-discharge follow-up, including referral to pulmonary rehabilitation when appropriate [13]. Our findings suggest that several of these recommended elements are inconsistently implemented in routine clinical practice. Although most respondents reported regularly arranging follow-up visits and frequently providing inhaler technique education, written action plans and comprehensive patient education were addressed less consistently. In addition, the low use of structured discharge summaries and limited multidisciplinary involvement indicate that many components of guideline-recommended transitional care remain insufficiently standardized. An additional future direction may involve integrating treatable traits and exacerbation-phenotyping concepts into structured discharge pathways. Current COPD discharge bundles primarily focus on standardized process-of-care elements, including treatment optimization, patient education, and follow-up planning (Miravitlles et al., 2023) [11]. However, emerging evidence suggests that exacerbations represent biologically distinct events, including eosinophilic, bacterial, viral, and pauci-inflammatory phenotypes, some of which appear to recur within individual patients and may have different therapeutic implications (Bafadhel et al., 2011) [24]. Furthermore, the treatable traits approach has been proposed as a strategy to move beyond traditional disease labels toward individualized management based on identifiable and modifiable clinical characteristics (Agusti et al., 2016) [25]. In the future, readily available markers such as blood eosinophil counts and inflammatory biomarkers could potentially support more personalized post-discharge treatment optimization and follow-up planning. However, further research is required before phenotype-guided discharge strategies can be routinely implemented in clinical practice, particularly given the existing challenges associated with the implementation of standard discharge protocols. Taken together, our findings suggest that improving COPD discharge practices requires addressing system-level barriers and facilitating the translation of existing knowledge into routine practice. Rather than increasing awareness, future efforts should focus on implementing simple, scalable, and standardized solutions. The SWOT analysis identified several potential areas for future implementation efforts, including discharge standardization, improved inter-level communication, and greater integration of structured discharge tools into routine clinical practice.

This study has several limitations.

First, as a survey based on self-reported practices, the findings may not fully reflect actual clinical behavior and may be subject to recall bias and social desirability bias. In addition, objective validation of reported practices through medical record review or clinical audit was not performed. Furthermore, the questionnaire did not undergo formal psychometric validation, including assessment of internal consistency or test–retest reliability.

Second, participation was voluntary, and recruitment through the Respiratory Society of Serbia may have favored physicians with a greater interest in COPD management and quality-improvement initiatives, introducing the possibility of selection bias. Responses were collected anonymously, precluding formal comparisons between respondents and non-respondents. The predominance of respondents from tertiary care institutions and the relatively short recruitment period may further limit generalizability, although the participation rate was high (approximately 71% of Society membership).

Third, the findings should be interpreted within the context of the surveyed physician network and healthcare setting. The survey focused specifically on discharge practices following hospitalization for COPD exacerbations and may not fully reflect management approaches for milder exacerbations treated exclusively in outpatient settings. The study did not collect clinical outcome data, including actual readmission rates, and therefore potential effects of structured discharge interventions on patient outcomes remain hypothetical. Finally, the SWOT analysis represented an interpretative synthesis of survey findings developed by the authors. It was not formally validated by external stakeholders, which may have introduced some degree of subjectivity.

Despite these limitations, the study provides valuable insights into current physician-reported COPD discharge practices and identifies areas where the implementation of guideline-recommended care can be strengthened.

This exploratory survey identified substantial variability in physician-reported COPD discharge practices and highlighted a gap between recognition of recommended discharge elements and their routine implementation in clinical practice. The findings suggest that barriers to implementation are predominantly organizational and system-related rather than attitudinal, as reflected in physicians’ high willingness to adopt structured discharge tools despite their limited routine use.

The results are specific to the Serbian healthcare setting and should be interpreted within the context of its organizational structure. Nevertheless, the identified challenges, including discharge standardization, inter-level communication, and coordination of post-discharge care, may also be relevant to other middle-income healthcare systems facing similar implementation barriers.

Overall, the findings represent an exploratory assessment of discharge practice patterns and may help identify priority areas for the future development and evaluation of structured discharge tools within this healthcare setting.

## 5. Conclusions

This physician survey demonstrates substantial variability in COPD discharge practices, despite high awareness of key elements of safe transitional care. A clear implementation gap exists between physicians’ recognition of best practices and their routine clinical application, driven primarily by system-level barriers and a lack of standardization. The findings highlight potential areas for improvement, particularly in patient education, multidisciplinary care, and discharge coordination. Importantly, physicians’ high willingness to adopt structured discharge summaries may facilitate future implementation efforts. As an exploratory, descriptive study, these findings should be interpreted in the context of the surveyed physician population and healthcare system. Nevertheless, they highlight priority areas for developing and evaluating structured discharge tools and implementation strategies to support more consistent transitional care for COPD.

## Figures and Tables

**Table 1:** Challenges reported by physicians as affecting safe discharge following hospitalization for COPD exacerbation.

Reported Challenges by Physicians that Affect Safe COPD Hospital Discharge	*n* (%)
Preventive measures and smoking cessation counseling	69 (69%)
Poor communication between healthcare levels	61 (61%)
Comorbidities and polypharmacy	60 (60%)
Social and logistical factors	60 (60%)
Inadequate patient and caregiver education	56 (56%)
Home oxygen therapy and non-invasive ventilation	35 (35%)
Lack of planned follow-up after discharge	25 (25%)
Insufficient assessment of clinical stability before discharge	16 (16%)
Unclear or incomplete discharge therapy	10 (10%)

**Table 2:** Physician-reported follow-up, patient education, and self-management support practices following COPD exacerbation.

Physician Reported Practice Following COPD Exacerbation	Always (%)	Often (%)	Sometimes (%)	Rarely (%)	Never (%)
Scheduled follow-up arranged before discharge	71	18	9	2	0
Inhaler technique education provided before discharge	33	40	24	2	1
Written action plan provided at discharge	7	19	19	31	24

**Table 3:** Factors perceived to contribute to COPD readmission following hospitalization for exacerbation.

Factors Perceived to Contribute to COPD Readmission Following a COPD Hospitalization	*n* (%)
Active smoking	90 (90%)
Poor treatment adherence	81 (81%)
Comorbidities	77 (77%)
Incorrect inhaler technique	72 (72%)
Inadequate social support	50 (50%)
Inadequate family support	50 (50%)
Limited access to therapy	45 (45%)
Inadequate patient education	41 (41%)
Absence of follow-up visits	28 (28%)

**Table 4:** Components recommended for inclusion in a structured COPD discharge summary.

Components Recommended for Inclusion in a Structured COPD Discharge Summary	*n* (%)
Current illness and hospital treatment course	96 (96%)
Lung function parameters	96 (96%)
Discharge pharmacological therapy	96 (96%)
Primary diagnosis and reason for admission	95 (95%)
Secondary diagnoses and comorbidities	95 (95%)
Laboratory and diagnostic findings	94 (94%)
Smoking cessation counseling	94 (94%)
Patient identification data	93 (93%)
Medical history and relevant prior findings	93 (93%)
Hospitalization details	91 (91%)
Treatment course and response	90 (90%)
Home oxygen therapy recommendation	90 (90%)
Vaccination status	90 (90%)
Follow-up plan	90 (90%)
Respiratory rehabilitation	88 (88%)
Comorbidity assessment	79 (79%)
Consultation reports	64 (64%)

**Table 5:** Perceived barriers to implementing a structured COPD discharge summary.

Perceived Barriers to Implementing a Structured COPD Discharge Summary	*n* (%)
Lack of a standardized institutional discharge template	70 (70%)
Insufficient time and increased workload	57 (57%)
Inadequate coordination between hospitals and primary care	47 (47%)
Resistance to changing established discharge practices	40 (40%)
Lack of awareness about structured discharge summaries	34 (34%)
Limited technical resources	28 (28%)

## Data Availability

The data supporting the findings of this study are not publicly available due to survey confidentiality and institutional restrictions governing data sharing but are available from the corresponding author upon reasonable request.

## Supplementary Materials

The following supporting information can be downloaded at: https://www.mdpi.com/article/10.3390/healthcare14121786/s1. The complete questionnaire:“Is a Structured Discharge Summary Needed After Hospital Treatment of Patients With COPD?”, including all items and response options, is provided as online data in Supplementary File S1.

## Abbreviations

The following abbreviations are used in this manuscript:

COPD	Chronic Obstructive Pulmonary Disease
GOLD	Global Initiative for Chronic Obstructive Lung Disease
SWOT	Strengths, Weaknesses, Opportunities, and Threats
GERD	Gastroesophageal Reflux Disease
SPSS	Statistical Package for the Social Sciences
