# Supplementary material for: Discharge Practices After Hospitalization for COPD Exacerbations: A Physician Survey and SWOT Analysis
Source: Healthcare (Basel). 2026 Jun 20;14(12):1786. doi: 10.3390/healthcare14121786 (PMC13300697; doi:10.3390/healthcare14121786)
Supplement: Supplementary file 1 [file healthcare-14-01786-s001.zip › healthcare-4338599-supplementary.pdf]

Supplementary Appendix 1. Survey Questionnaire

Title: Is a Structured Discharge Summary Needed After Hospital Treatment of Patients With COPD?

The questionnaire was originally developed and administered in Serbian and translated into English for publication.

Q1. Your current professional role (select one):
- Pulmonology subspecialist
- Internal medicine specialist working in a pulmonology department
- Pneumophthisiology specialist
- Internal medicine resident working in a pulmonology department
- General physician working in a pulmonology department

Q2 Are you working in the:

-Primary care Institution

-Secondary Care Institution

-Tertiary Care Institution

-Private Practice

Q3. Which members of the healthcare team are usually involved in preparing a COPD patient for discharge from the hospital? (Select all that apply)
- Specialist
- Resident
- General physician
- Nurse
- Respiratory therapist
- Pharmacist
- Physiotherapist
- Social worker

Q4. I currently use a structured discharge summary for patients hospitalized due to COPD exacerbation (A discharge summary is an important clinical documentation that accurately

and succinctly describes the patient's medical history, diagnoses, treatment and follow-up plans during hospital admission).

- Never
- Rarely
- Sometimes
- Often
- Always

Q5. The discharge summary and discharge process after COPD exacerbation are standardized in my team/department/institution.
- Never
- Rarely
- Sometimes
- Often
- Always

Q6. In my institution, there is a person responsible for coordinating the COPD patient discharge process.

- Never
- Rarely
- Sometimes
- Often
- Always

Q7. What are the most common challenges you face that should be addressed to ensure safe discharge of patients after a COPD exacerbation? (Select all that apply)
- Inadequate assessment of clinical stability at discharge
- Inadequate patient and caregiver education
- Incomplete or unclear discharge therapy
- Lack of a follow-up plan
- Poor communication across healthcare levels
- Comorbidities and polypharmacy
- Social and logistical factors
- Preventive measures and smoking cessation counseling
- Home oxygen therapy and non-invasive ventilation
- Other

Q8. Patients are given clear information about their scheduled follow-up visit before being discharged after a COPD exacerbation.

- Never
- Rarely
- Sometimes
- Often
- Always

Q9. Cardiovascular comorbidities (arterial hypertension, coronary artery disease, heart failure, arrhythmias, pulmonary hypertension, peripheral arterial disease) are adequately considered at discharge of COPD patients

- Never
- Rarely
- Sometimes
- Often
- Always

Q10. Other common comorbidities in COPD patients (lung cancer, osteoporosis, anxiety/depression, sarcopenia, GERD, metabolic diseases) are adequately considered at discharge.
- Never
- Rarely
- Sometimes
- Often
- Always

Q11. Patients are provided with comprehensive counseling and training on correct inhaler use prior to discharge.
- Never
- Rarely
- Sometimes
- Often
- Always

Q12. Patients receive a written action plan for managing future COPD exacerbations at discharge.
- Never
- Rarely
- Sometimes
- Often
- Always

Q13. Patients clearly understand the instructions they receive from physicians upon hospital discharge.
- Strongly disagree
- Disagree
- Neutral
- Agree
- Strongly agree

Q14. A significant number of COPD patients are re-hospitalized within 30 days after a previous exacerbation.
- Strongly disagree
- Disagree
- Neutral
- Agree
- Strongly agree

Q15. A structured discharge summary can help reduce rehospitalizations after a COPD exacerbation.
- Strongly disagree
- Disagree
- Neutral
- Agree
- Strongly agree

Q16. Which factors do you consider to contribute most to COPD rehospitalization? (Select all that apply)
- Absence of follow-up visits
- Poor treatment adherence
- Comorbidities
- Inadequate patient education
- Incorrect inhaler technique
- Insufficient family support
- Inadequate social support
- Limited access to therapy
- Active smoking
- Other

Q17. A structured discharge summary after COPD exacerbation would improve the quality of care.
- Strongly disagree
- Disagree
- Neutral
- Agree
- Strongly agree

Q18. Which components should a structured discharge summary include? (Select all that apply)
- Patient identification data
- Hospitalization details
- Primary diagnosis/reason for admission
- Secondary diagnoses and comorbidities
- Medical history and relevant prior findings
- Current illness and hospital treatment course
- Laboratory and diagnostic findings
- Lung function parameters
- Consultation reports
- Comorbidity assessment
- Treatment course and response
- Prescribed discharge therapy
- Assessment of home oxygen therapy needs
- Smoking cessation counseling
- Vaccination status
- Respiratory rehabilitation
- Patient education
- Follow-up plan
- Referral for further diagnostics
- Hospital/physician contact information
- Physician signature and institutional stamp
- Other

Q19. "How likely are you to utilize a structured COPD discharge summary in your practice? (1 = not at all likely; 10 = extremely likely)

Q20. What do you consider the main barriers to implementing a structured discharge summary? (Select all that apply)
- Lack of time and increased administrative burden
- Absence of a unified, standardized institutional discharge letter

- Limited technical resources
- Insufficient education and awareness about the structured discharge concept
- Resistance to changing established discharge practices
- Poor coordination and communication between hospitals and primary care institutions

-Other
